# *Progeny Clustering*: A Method to Identify Biological Phenotypes

**DOI:** 10.1038/srep12894

**Published:** 2015-08-12

**Authors:** Chenyue W. Hu, Steven M. Kornblau, John H. Slater, Amina A. Qutub

**Affiliations:** 1 Department of Bioengineering, Rice University; 2 Departments of Leukemia and Stem Cell Transplant, University of Texas MD Anderson Cancer Center; 3 Department of Biomedical Engineering, University of Delaware

## Abstract

Estimating the optimal number of clusters is a major challenge in applying cluster analysis to any type of dataset, especially to biomedical datasets, which are high-dimensional and complex. Here, we introduce an improved method, *Progeny Clustering*, which is stability-based and exceptionally efficient in computing, to find the ideal number of clusters. The algorithm employs a novel *Progeny Sampling* method to reconstruct cluster identity, a co-occurrence probability matrix to assess the clustering stability, and a set of reference datasets to overcome inherent biases in the algorithm and data space. Our method was shown successful and robust when applied to two synthetic datasets (datasets of two-dimensions and ten-dimensions containing eight dimensions of pure noise), two standard biological datasets (the Iris dataset and Rat CNS dataset) and two biological datasets (a cell phenotype dataset and an acute myeloid leukemia (AML) reverse phase protein array (RPPA) dataset). *Progeny Clustering* outperformed some popular clustering evaluation methods in the ten-dimensional synthetic dataset as well as in the cell phenotype dataset, and it was the only method that successfully discovered clinically meaningful patient groupings in the AML RPPA dataset.

Cluster analysis, one of the most useful unsupervised learning techniques in the era of big data, has been widely applied in biomedical research^1^,^2^. Biological datasets are often large, high-dimensional and noisy, and prior knowledge of the underlying distribution is usually lacking. Clustering in this case could provide key insights into the data by automatically organizing the data into groups of distinct patterns. With the field of genomics flourishing during the last two decades, cluster analysis has been extensively applied to the analysis of gene expression profiles across time, tissue samples and patients^3^,^4^,^5^,^6^. In particular, tumor classification is one of the hottest application fields, in which tumor classes based on different gene expression patterns and survival outcomes may help in the design of better targeted therapies^7^,^8^,^9^,^10^. With recent advances in systems biology and high-throughput technology, we envision an increasing need and broader application potential for cluster analysis in biomedical research. For example, the identification and categorization of cell phenotypes based on quantitative imaging metrics, as we will introduce later, is one of these emerging areas for applying cluster analysis.

A major challenge in cluster analysis is finding the optimal number of clusters^11^,^12^. Unfortunately, the inherent number of clusters is most often unknown to researchers. Though some clustering methods are able to automatically determine the number of clusters (e.g. *Self-Organizing Maps*^13^, *Affinity Propogation*^14^), most clustering algorithms (including the popular clustering methods *k-means*^15^ and *hierarchical clustering*^16^) require input from users to specify the number of clusters. In *k-means*, the number of clusters needs to be given pre-clustering to initiate the algorithm, whereas in *hierarchical clustering* a cutoff for the dendrogram needs to be specified post-clustering.

An increasing effort has been made in the last two decades to design an objective measure of how well data are clustered into various numbers of groups, which transforms the cluster number determination into a model selection problem^17^,^18^. Most of the methods employ either distance-based or stability-based measures. Distance-based methods, such as *Gap Statistics*^19^ and *Silhouette*^20^, evaluate the quality of clustering by measuring the within-cluster distance and the cross-cluster distance. The main assumption is that a good clustering should produce close proximity among observations within each cluster and sufficient separation between observations in different clusters. Though distance-based approaches are easily implementable and computationally efficient, their model-dependent nature restricts their application to distance-based clustering only, and their dependence on absolute distance can hinder their effectiveness when applied to high-dimensional data. Stability-based methods, such as *Clest*^21^, *Consensus Clustering*^22^ and *Model Explorer*^23^, approach clustering quality from a rather different angle. The philosophy behind them resonates with the popular concept of *Stability Selection*^24^. Assuming that observations are sampled from a fixed but unknown population, when samples are repetitively drawn from the same population, the clustering solution should not vary drastically. Instead of directly measuring cluster compactness and separation, stability-based methods evaluate how robust the clustering is against the randomness in sampling. Methods under this category were found to perform robust in practice, but they are slow to compute^25^, considering the repetitive nature of the algorithm. The computation costs can dramatically escalate when we encounter big data, since the computational complexities of almost all clustering algorithms are dependent on the data size.

Here, we introduce a biologically inspired approach, *Progeny Clustering*, to estimate the ideal number of clusters. Our method is based on fundamental notions in stability analysis^26^,^27^, especially on Levine and Domany’s approach^28^, which first assigns cluster membership to the full dataset and then validates clustering consistencies among resampled subsets. Their approach avoids selecting and applying classifiers, but is criticized for reusing the same samples for validation, which could lead to overestimation of cluster stability. Our *Progeny Clustering* method employs a novel sampling technique, *Progeny Sampling*, which constructs new samples out of existing ones by sampling features independently. This approach not only avoids reusing the same samples but also reduces data size for re-clustering. Since feature independence is assumed in *Progeny Sampling*, caution should be given when applying the method to data with dependent features, and dimensional reduction techniques are recommended to first transform the data into orthogonal feature space. The measure of stability used in *Progeny Clustering* is based on a co-occurrence probability matrix that captures true classification and false classification when new samples are repetitively drawn and clustered. Reference datasets, similar to those used in *Gap Statistics*, are employed to overcome potential biases inherent in the algorithm or data space.

We first validated the performance of *Progeny Clustering* using two synthetic datasets, which showed that the method is stable and robust against varied sampling sizes, additional noisy dimensions and noise present in important dimensions of the data. We then examined its performance on two commonly used biological datasets to showcase the strength of the method as well as to offer a glimpse into how to choose the optimal cluster number using different criteria. Finally, we applied *Progeny Clustering* to analyze two new biological datasets: a cell phenotype dataset and an Acute Myeloid Leukemia (AML) reverse phase protein array (RPPA) dataset. The method was successful in correctly identifying phenotype groups from cell images, and it was effective in discovering clinically meaningful patient groups based on their protein expression levels. Furthermore, we also illustrated the computational advantage of *Progeny Clustering* compared to other stability-based methods using the AML RPPA data.

## Methods

In this section, we describe the *Progeny Clustering* algorithm step by step. The mathematical descriptions of other popular cluster evaluation methods implemented in this study are detailed in the Supplement.

Let {*x*_*ij*_}, 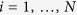, 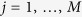, be a finite dataset on *M* features (e.g., protein expression levels, phenotyping metrics) for *N* independent observations (e.g., AML patients, cells). Suppose we have a clustering method (e.g., *k-means*) that partitioned the data into *K* clusters, *C*_1_, …,*C*_*K*_. As cluster analysis groups observations that share similar characteristics and distinguishes those that do not, ideally each cluster *C*_*k*_,  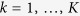, would have a distinct characteristic or be compact in space. Therefore, we assume that the whole population is heterogeneous and can be inherently divided into *K* more homogeneous subpopulations. Then, each observation in 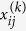 can be viewed as being randomly sampled from a subpopulation corresponding to the cluster it belongs to (*C*_*k*_).

In contrast to traditional sampling methods that operate on the entire dataset for reclustering, *Progeny Clustering* employs a new sampling approach to exploit the inherent heterogeneity of the population as well as to reduce the computation costs of the entire analysis. In essence, it samples values from each feature individually to construct new imaginary samples within each cluster. We call these new imaginery samples *Progenies* and this process *Progeny Sampling*. Let 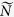 (e.g., 5, 10, 20) be the number of Progenies sampled for each cluster *C*_*k*_. Our new validation dataset 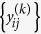, 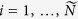, 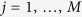,  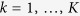, then has a total size of 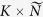 observations with *M* features. To construct each 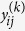 from *C*_*k*_, a sample is randomly drawn from the *j*^*th*^ feature in *C*_*k*_. As the location, the span and the density of each feature space are characteristic of each cluster and somewhat different from that of other clusters, the *Progeny Sampling* allows us to assess the distinctness, homogeneity and compactness of each cluster without using the same samples and enables us to reduce the sample size for validation (as shown later in Results).

The new observations constructed from each cluster 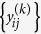 will be combined into one new dataset {*y*_*ij*_} and clustered using the same method as is used when clustering the original dataset {*y*_*ij*_}. The clustering assignments will be represented in a 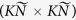 co-occurrence matrix *Q*, with


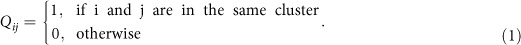


The co-occurrence matrix is symmetric, i.e. *Q*_*ij*_ = *Q*_*ji*_. Furthermore, the co-occurrence matrix is arranged in such a way that observations reconstructed from the same original cluster are next to each other, i.e. 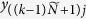 to 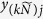 are sampled from *C*_*k*_. The co-occurrence matrix thus can be divided into two regions: *K* blocks of true classification along the diagonal, and (*K* − 1) × *K* blocks of false classification, in which each block is of size 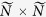. If there is absolute agreement between the new and the original clustering assignments, *Q* would be a perfect block-diagonal matrix of *K* non-overlapping blocks of 1 s along the diagonal, surrounded by blocks of 0 s.

If we repetitively construct new datasets and perform cluster analysis *R* times, we would obtain a series of co-occurrence matrices. Each co-occurrence matrix is denoted as *Q*^(*r*)^, 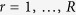. To summarize *Q*^(*r*)^ from each repetition, we define a co-occurrence probability matrix *P*:


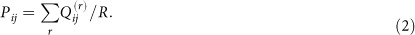


The co-occurrence probability matrix *P* has the same property as *Q*^(*r*)^, consisting of *K* blocks of true classification likelihood along the diagonal and (*K* − 1) × *K* blocks of false classification likelihood in the rest of the matrix. The more robust and stable the clustering is, the higher ratio of true classification vs. false classification there will be. We therefore define a score for clustering stability as


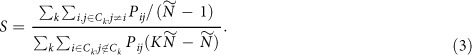


**Figure 1:**
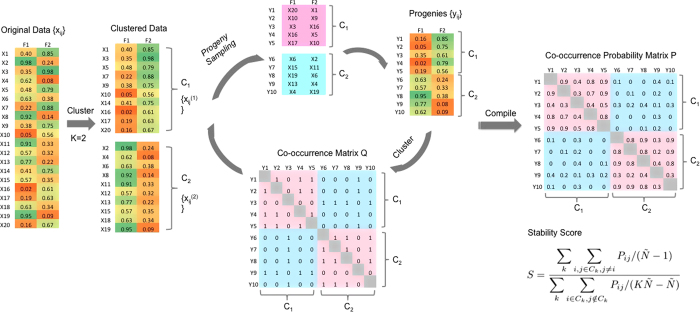
An illustration of the core steps in *Progeny Clustering*. The dataset in the example consists of 20 samples (denoted as *X*) in a two-dimensional space (denoted as *F*1 and *F*2). The scheme displays the workflow of generating a stability score for clustering this dataset into two clusters. Five progenies (denoted as *Y*) were generated for each cluster. The co-occurence matrix *Q* represents one of the clustering results of the mixed progenies, in which matrix entries are 1 if two progenies are in the same cluster and 0 otherwise. In both co-occurrence matrices *Q* and *P*, the true classification region containing progenies from the same initial cluster is colored pink and the false classification region containing progenies from different initial clusters is colored light blue. If the clustering quality is high, we would expect more 1 s (*Q*) or higher probabilities (*P*) in the true classification region and more 0 s (*Q*) or lower probabilities (*P*) in the false classification region.

The core algorithm of *Progeny Clustering* is described below. An example using a toy dataset is illustrated in Fig. 1. The parameter values used in this paper are shown in Table 1. For consistency, we used *k-means* as the clustering algorithm to couple with *Progeny Clustering* and other competing techniques in application to all datasets and experiments in this study. *Hierarchical clustering* using ward linkage was used in application to synthetic datasets as a proof-of-concept to show the capability of *Progeny Clustering* working with clustering algorithms besides *k-means*.


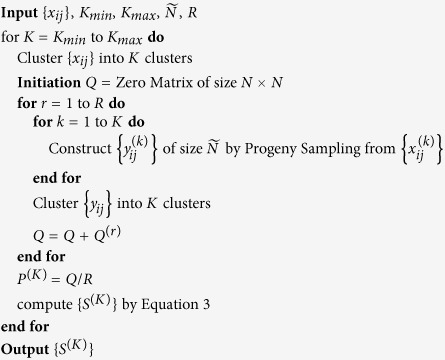


To minimize biases inherent in the dataset and algorithm, reference datasets that are randomly sampled from the same data space will serve as the control. The reference dataset 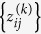 can be generated either from a uniform distribution over the range of each feature or from a uniform distribution over a box aligned with the principle components of the data. The former is used here for its simplicity. *T* reference datasets will be generated by Monte Carlo simulation, and each of them will be treated with the same core algorithm performed on the original dataset with output denoted as 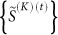, 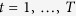. The difference in score at each number of clusters when comparing *S* to 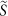 is


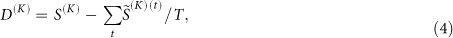


where 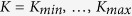.

To choose the optimal number of clusters *K*_*o*_, we can use either one of the following criteria:


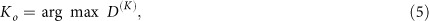



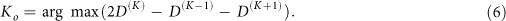


**Figure 2:**
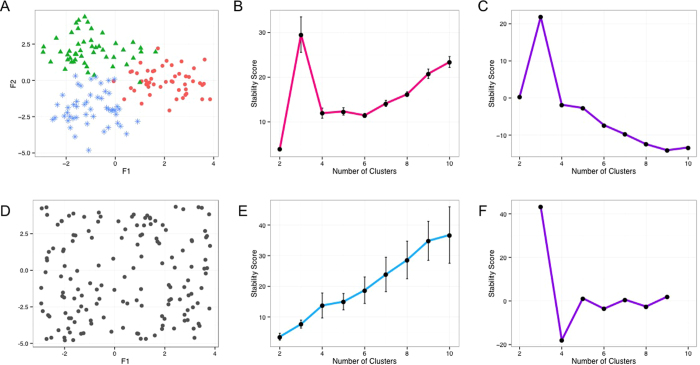
An illustrative example of applying *Progeny Clustering* to a three-cluster two-dimensional toy dataset: (**A**) a scatterplot of the original dataset showing its three-cluster structure; (**B**) the stability score curve of the original dataset illustrating the clustering quality of the data from 2 clusters through 10 clusters; (**C**) the stability curve generated by Equation (4) based on the “greatest score” criterion suggesting three as the optimal cluster number; (**D**) a scatterplot of an example reference dataset showing lack of cluster structure; (**E**) the stability score curve of the reference datasets showing that the clustering stability linearly increases with an increase in cluster number; (**E**) the stability curve generated by Equation (4) based on the “greatest score” criterion suggesting three as the optimal cluster number; (**F**) the stability curve generated by Equation (7) based on the “greatest gap” criterion suggesting three as the optimal cluster number.

The “greatest score” criterion defined in Equation (5) selects the cluster number that generates the greatest stability score among all examined numbers, whereas the “greatest gap” criterion defined in Equation (6) looks for local gaps in stability scores. Specifically the “greatest gap” criterion selects the cluster number that renders the greatest difference in stability score compared to its neighboring numbers. We are proposing two criteria here, not only because they are equally intuitive and rational, but also because they can serve different application needs. Compared to the “greatest score” criterion, the drawback of the “greatest gap” is that it is unable to assess the clustering quality at *K*_*min*_ and *K*_*max*_. On the other hand, since we observed a linear relationship between cluster numbers and stability scores in the reference datasets (Fig. 2D), Equation (6) can be simplified to Equation (7), which only involves computing stability scores of the original dataset. The unique property of the “greatest gap” criterion allows us to greatly cut down computational costs involved in generating reference stability scores. The performances of both criteria are shown and discussed later in Results.


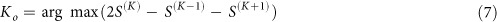


## Results

### Performance Testing Using Synthetic Datasets

We first applied our algorithm to a toy dataset to illustrate how it works. The toy dataset contains three clusters centered around (−1,2), (2,0) and (−1,−2) in a two-dimensional space (Fig. 2A). Each cluster consists of 50 samples that were drawn from bivariate normal distributions with a common identity covariance matrix. An example of reference datasets generated from a uniform distribution over the range of each feature is shown in Fig. 2D, in which no inherent pattern or organization is visible. The stability score curves for the original and reference datasets are shown in Fig. 2B,E. The standard deviation of the score for the original dataset (computed by iterating the algorithm 25 times) was found to be small, indicating that the algorithm is fairly stable. Here, we computed the standard deviation to investigate the stability of the algorithm, and this exercise is not deemed necessary for real applications. The stability scores from the reference dataset displayed a linear relationship with the cluster numbers, fitting well our expectation of applying *Progeny Clustering* to a random dataset. In datasets without inherent organization like the reference datasets, higher cluster numbers are usually more preferable, since they build up stronger cluster identities by splitting the dataset into smaller pieces. This linear property is computationally favorable when using the “greatest gap” criterion to determine the best cluster number, since the score gaps between each cluster number and its neighboring numbers get canceled out, resulting in values close to zero at all cluster numbers. To determine the number of clusters, we generated the “greatest score” curve by subtracting the mean stability scores of reference datasets from that of the original dataset (Fig. 2C) and the “greatest gap” curve by subtracting the stability score at each cluster number by its neighboring stability scores (Fig. 2F). Both criteria clearly suggested three as the optimal cluster number for this toy dataset. We repeated this experiment ten times, and the algorithm identified the same cluster number in each toy dataset generated.

**Figure 3:**
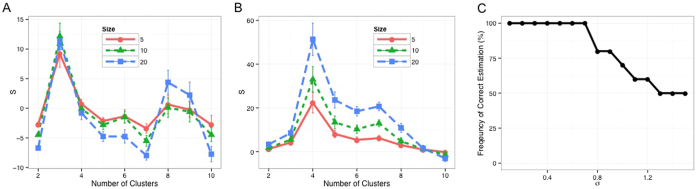
Robustness of *Progeny Clustering* against varied sampling sizes (5, 10 and 20 progenies generated per cluster) in the (**A**) two-dimensional and (**B**) ten-dimensional toy datasets. In both cases, the three curves derived using different sampling sizes were consistent in trends, which indicates the algorithm’s robustness. (**C**) Sensitivity of *Progeny Clustering* to noise present in the three-cluster, two-dimensional dataset. Noise was generated with *σ* ranging from 0.1 to 1.5, and the frequency of correct estimation was calculated based on ten repeated experiments.

We then explored how robust the algorithm is against varied sampling sizes and whether there is a lower limit for the size of *Progenies*. Answering this question can assist us in selecting a reasonably small size for *Progeny Sampling* and subsequent re-clustering, which directly relates to the computation costs of the algorithm. Figure 3A shows the *Progeny Clustering* curves for sampling sizes of 5, 10 and 20 in the same two-dimensional toy dataset we used before, and all of them output a highest score for the three-cluster partition. Besides agreement on the optimal number of clusters, the three curves are almost identical to each other, which further increases our confidence in the reliability and robustness of the algorithm.

It is known that conclusions drawn from statistical analyses can be unreliable and biased when a small sample size is coupled with high dimensions. We wondered if a small sampling size remains effective when applied to a dataset of higher dimensions. Hence, we created a ten-dimensional toy dataset consisting of four clusters (50 samples each) to test the algorithm’s robustness against additional noisy dimensions. The first two dimensions were sampled from bivariate normal distributions centered at (4, 4), (4, −4), (−4, 4) and (−4, −4) with a common identity covariance matrix, while the rest of the eight dimensions are noises sampled from a standard normal distribution. The same test was performed on this ten-dimensional dataset with sampling sizes of 5, 10 and 20 (Fig. 3B). Similar to what we have observed in the two-dimensional dataset, all of the three *Progeny Clustering* curves consistently produced a highest score for partitioning the data into four groups, in agreement with the initial design of this dataset. It is worth noticing that sample sizes as small as 5, much smaller than the number of dimensions (10), were sufficient to make the algorithm work properly. When we compared the performance of our algorithm to other popular methods (Table 2), we were surprised to find that several stability-based methods (e.g., *Clest*, *Consensus Clustering*) failed to identify the optimal number of clusters in this ten-dimensional toy dataset, though nearly all of them worked well in the previous two-dimensional toy dataset. We saw consistent results when we repeated this experiment ten times.

In addition to using noisy dimensions, we also examined the sensitivity of *Progeny Clustering* to noise added on top of the real dimensions. Based on the three-cluster, two-dimensional dataset that we used previously, we added noise generated from a Gaussian Distribution (*μ* = 0) and scaled *σ* from 0.1 to 1.5 to investigate the break point of the algorithm. We repeated the experiments ten times and found that *Progeny Clustering* is robust against a decent amount of noise present in the real dimensions (Fig. 3C).

To showcase the performance of *Progeny Clustering* using clustering algorithms other than *k-means*, we applied *Progeny Clustering* coupled with *hierarchical clustering* using *ward linkage* to both the three-cluster, two-dimensional dataset and the four-cluster, ten-dimensional dataset. The results obtained using *hierarchical clustering* are consistent with what we observed using *k-means* (Figure S8). In both cases, *Progeny Clustering* was able to identify the correct number of clusters.

### Performance Testing Using Standard Biological Datasets

We then tested the performance of *Progeny Clustering* using two standard biological datasets, the Iris dataset^29^ which can be downloaded from the UCI machine learning repository^30^ and the Rat Central Nervous System (CNS) dataset^31^. Both of these datasets have been frequently used to test and evaluate clustering techniques. The Iris dataset contains 50 samples from each of three species of Iris (*Iris setosa*, *Iris virginica* and *Iris versicolor*) with four features measured from each sample (the length and width of the sepals and petals). Though the Iris dataset contains three species, the number of two clusters is commonly accepted as the golden standard due to the limited capability of the four features to distinguish *Iris virginica* from *Iris versicolor*. The Rat CNS dataset consists of expression profiles of 112 genes over 17 conditions during rat central nervous system development. Though no prior knowledge of true cluster number is available for this dataset, we took six clusters suggested in the initial study to be the gold standard, which has also been used in other clustering studies^25^.

**Figure 4:**
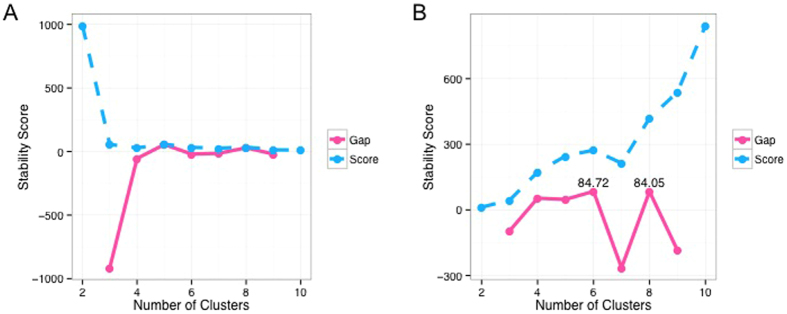
The *Progeny Clustering* results for the (**A**) Iris dataset and (**B**) Rat CNS dataset. Curves from both criteria were shown, indicating two or five clusters in (**A**) and six or ten clusters in (**B**).

The clustering evaluation results from *Progeny Clustering* for the Iris dataset and the rat CNS dataset are shown in Fig. 4A,B respectively. We observed that in both of these cases, there is discrepancy of the optimal cluster number picked by the two criteria (“greatest score” and “greatest gap”). This is actually a good example to showcase in real application the pros and cons of each criterion. In application to the Iris dataset, the “greatest score” criterion accurately estimated two as the optimal cluster number, whereas the “greatest gap” criterion failed because it was unable to evaluate clustering quality at the minimum cluster number tested. On the other hand, the “greatest gap” criterion correctly identified six as the optimal cluster number for the Rat CNS dataset, while the “greatest score” criterion incorrectly suggested ten. Since the stability score curve based on the “greatest score” criterion resembles the typical linear score curve generated from reference datasets, we advise not to directly pick the maximum cluster number when it is associated with the greatest stability score. In this case, either cluster numbers greater than the maximum cluster number should be tested or the “greatest gap” curve should be consulted.

### Application to Identifying Cell Phenotypes

**Figure 5:**
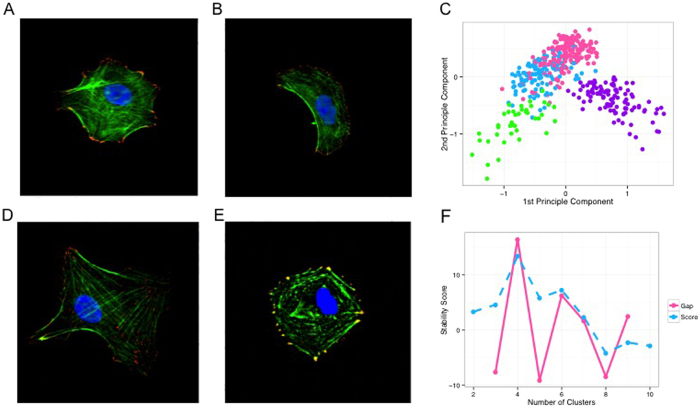
Images of four main categories of cell phenotypes: (**A**) star-shaped (**B**) crescent-shaped; (**D**) Texas-shaped; (**E**) square-shaped. In each of these images, cells were fluorescently immunolabeled for the cytoskeletal proteins vinculin and actin, and stained by DAPI for nuclei. Image analysis results: (**C**) a scatterplot of the four cell categories in the space of the first two principle components, in which each dot represents a cell and the dot’s color indicates its cell type. This shows that the features we used to characterize the cell phenotypes are able to distinguish the four categories. (**F**) *Progeny Clustering* curves indicating four as the optimal number for clustering.

To demonstrate how *Progeny Clustering* can be applied to identify biological phenotypes, we used it to estimate the number of cell phenotypes captured in a cell imaging dataset. A description of the dataset and the experiment design is detailed in the Supplement. Briefly, 440 cells were cultured on arrays of pattern configurations derived from 4 cells of interest (COIs) that were created via Image Guided Laser Scanning Lithography^32^, a variant of Laser Scanning Lithography^33^ that uses images to define pattern configurations, resulting in 4 major groups of cells with distinct cytoskeletal and morphological phenotypes (Fig. 5A,B,D,E). The phenotype of each cell was then captured and quantified in 41 morphology metrics, resulting in a dataset consisting of 444 samples in 41 dimensions with 4 centers.

Since many features (imaging metrics) in this dataset overlap in characterization and are highly correlated, we performed *Principle Component Analysis (PCA)* to capture metrics with most variance and to render an independent feature space. Figure 5C illustrates the distribution of the data within the first two principal components, in which four major clusters are recognizable. We then performed *Progeny Clustering* on the first three principle components of the data to estimate the inherent number of clusters (Fig. 5F). The *Progeny Clustering* curve indicates four inherent clusters of cells, since both criteria output curves peak at *K* = 4. This is in agreement with the inherent structure of the dataset (Fig. 5E) as well as the experiment design. In contrast, we saw some clustering evaluation methods failed to recognize the four-cluster structure using the same dataset (Table 2).

### Application to Identifying Leukemia Subclasses

**Figure 6:**
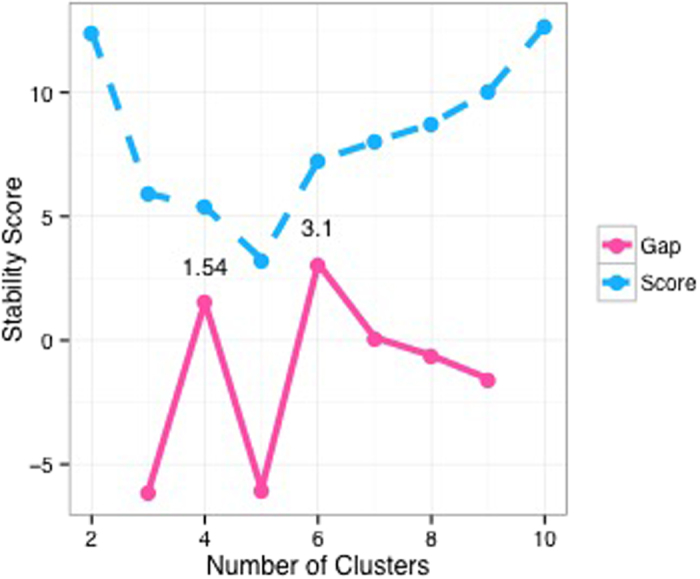
*Progeny Clustering* results for the AML RPPA dataset, suggesting 6 (“greatest gap”) or 10 (“greatest score”) as the optimal number of clusters.

Since one of the hottest application areas of clustering techniques is classifying cancerous tissues, we used a proteomic dataset to investigate the capability of *Progeny Clusteirng* to identify clinically meaningful subclasses in leukemia. The detailed description of the data preparation procedure can be found in the Supplement and the related reference^34^. Breifly, it is a dataset on expression levels of 10 adhesion-related proteins from 560 Acute Myeloid Leukemia (AML) patient samples collected by the University of Texas MD Anderson Cancer Center. Since AML is notorious for its heterogeneity^35^,^36^, the ability to classify patients based on their protein expression profiles can greatly benefit personalized medicine and tackle this heterogeneity. The result of applying *Progeny Clustering* to this dataset is shown in Fig. 6. In this case, the two criteria for selecting the optimal cluster number differ: the “greatest score” criterion suggests ten as the optimal number of clusters, whereas the “greatest gap” criterion suggests six as the optimal number of clusters.

**Figure 7:**
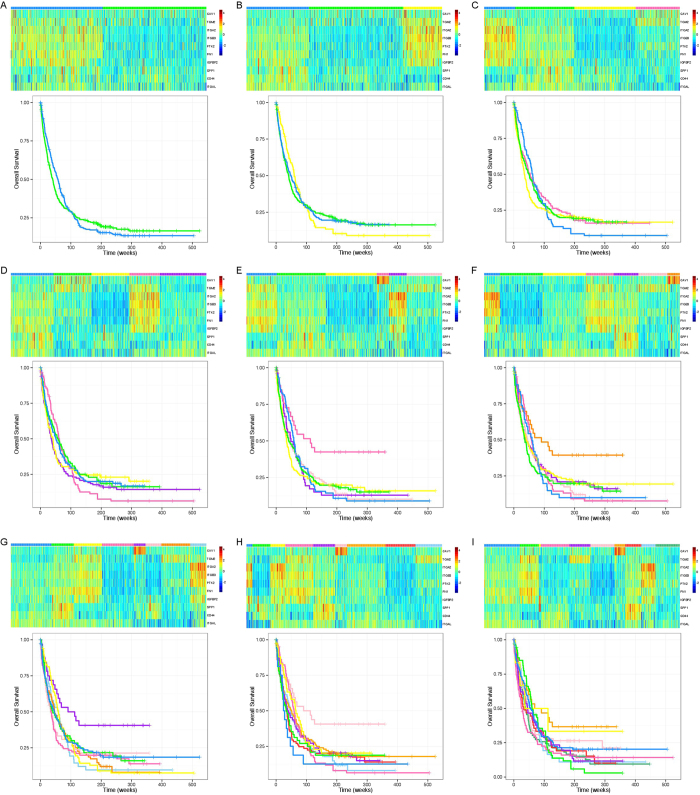
Heat-maps and overall survival curves for partitioning the AML patient adhesion pathway RPPA data into (**A**) 2; (**B**) 3; (**C**) 4; (**D**) 5; (**E**) 6; (**F**) 7; (**G**) 8; (**H**) 9 and (**I**) 10 clusters. Each cluster is represented by a unique color shown in the top bar of each heat-map. The same color for each cluster is used in both the heat-map and the survival curves.

To interpret and evaluate the performance of *Progeny Clustering* on this dataset, we plotted a series of cluster heat-maps to visualize the clustering quality (Fig. 7). A compact and homogeneous group featuring high levels of CAV1 first appeared in the 6-group partition (Fig. 7E) and persistently showed up afterwards (Fig. 7F–I). Similarly, a compact cluster featuring high levels of ITGA2 and PTK2 came into view in the 9-group partition (Fig. 7H), and another cluster featuring high levels of IGFBP2 and SPP1 emerged in the 10-group partition (Fig. 7I). As *Progeny Clustering* favors more compact and homogeneous clusters, the patterns observed in the heat-maps are well reflected in the score curve (Fig. 6). The score difference first leaps up when the number of clusters goes from 5 to 6. It then remains relatively the same from 6 to 8, and climbs up again at 9 and 10 due to the emergence of two new compact clusters.

To investigate the clinical significance of different clustering numbers, we looked at the survival outcomes of each classification (Fig. 7). The survival curves under 6 (Fig. 7E) and 10 (Fig. 7I) clusters, suggested by our *Progeny Clustering* algorithm, are particularly interesting when compared with other partitions, as a new major favorable group showed up in each of them. In comparison with other popular evaluation methods (Table 2), *Progeny Clustering* is the only one that successfully identified the number of six or ten as the ideal number of clusters, which is consistent with the patterns observed in the heat-maps and is of particular significance to clinical outcome.

**Figure 8:**
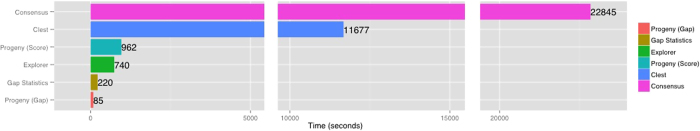
Computation time of *Progeny Clustering* using “greatest gap” criterion, *Gap Statistics*, *Model Explorer*, *Progeny Clustering* using “greatest score” criterion, *Clest* and *Consensus Clustering* in application to the AML RPPA dataset. Algorithms are ordered based on their computation speed.

The relatively large size of this dataset allows us to examine the computation costs of *Progeny Clustering* and compare its performance metrics to that of other algorithms. The performances of all algorithms except *Silhouette* are shown in Fig. 8. *Silhouette* was left out of this evaluation because the method does not perform any type of repetitive validation, thus is not reasonable to compare against. We used the same parameters (e.g., number of repetitions and parameters used in *k-means*) when testing each algorithm and kept the running environment the same to our best knowledge. The running time of *Consensus Clustering* and *Clest* is at a completely different scale from that of the other three algorithms, similar to what was seen previously in other comparative studies^25^. *Progeny Clustering* using the “greatest gap” criterion requires the least computation time, and is much faster than using the “greatest score” criterion due to the omission of generating reference stability scores.

## Discussion

We have developed an improved stability-based method, *Progeny Clustering*, to evaluate the quality of clustering and estimate the ideal number of clusters. Our method was shown successful when applied to two simulated datasets and robust with small sampling sizes and moderate level of noise. We then demonstrated its application potential to biomedical research by applying it to two standard biological datasets and two new biological datasets. In particular, our method outperformed some of the most popular evaluation methods in the high-dimensional toy dataset and in the biological datasets. Furthermore, our method is the only method that successfully identified the clinically meaningful partitions of patient groups in the AML RPPA dataset.

One of the novelties in the *Progeny Clustering* algorithm is the *Progeny Sampling*. In contrast to the traditional sampling scheme that preserves the exact identity of each sample, our approach draws features independently to construct imaginary samples that are representative of each cluster but are non-existent in the original dataset. This enables us to avoid reusing the same samples for validation, which theoretically undermines the effectiveness of the whole algorithm. The impetus behind it stems from the interest in finding trustworthy progenies in biomedical datasets, e.g., in the engineered cell population. As different numbers of clusters establish different progenies in the population, a good clustering in a biologist’s eye would generate robust progenies that well represent key characteristics of particular subpopulations. The *Progeny Sampling* method assumes that variation in one feature is independent from that in another, thus it decouples the relationship between features and easily exploits the fuzzy space around the progeny for cluster reconstruction. While this approach fits perfectly with centroid-based clustering algorithms (e.g., *k-means*), the coupling of *Progeny Clustering* with other clustering techniques or datasets that rely on inter-feature associations should proceed with caution. One potential solution to applying *Progeny Clustering* to datasets with dependent features is to first transform the raw data into reduced independent or orthogonal feature space using techniques such as PCA prior to the analysis. Since *Progeny Clustering* in this study was mainly tested using *k-means* and *hierarchical clustering*, further testing is merited to assess how well *Progeny Clustering* performs with clustering methods not considered in this study, as well as how *Progeny Clustering* fits in a clustering ensemble approach^37^.

Another advantage of using *Progeny Clustering*, besides its excellent performance, is its computational efficiency. Clustering is usually the most time consuming step in almost all classical clustering evaluation methods, the computation time of which increases with an increase in the sample size. For example, the complexity of *k-means* is *O*(*tkN*), and *hierarchical clustering* has complexity *O*(*N*^2^*logN*) for average and complete linkage^1^, where *k* is the number of clusters, *t* is the number of iterations, and *N* is the sample size of the dataset. The iteration of sampling and clustering, which is the essence of stability analysis, can magnify the effects of sample size and escalate the computation costs. In most stability-based methods, sampling is done at the scale of the entire dataset (e.g., *Consensus Clustering*) or at least at half of the dataset (e.g., *Clest*, *Model Explorer*). Thus, the evaluation task can be daunting, especially when it comes to biological datasets of huge sizes. *Progeny Clustering* can easily overcome this barrier, because the sampling size was designed to be independent of the original data size and the algorithm was demonstrated to tolerate small sampling sizes for validation. In addition, the unique linear property of the stability scores in reference datasets has enabled us to greatly accelerate the algorithm using the “greatest gap” criterion, since the time-consuming step of computing stability scores for reference datasets can be skipped. The algorithm was shown to perform at a completely different and faster scale compared to other stability-based methods.

The choice of parameter values for the *Progeny Clustering* algorithm is quite flexible. For instance, smaller sampling sizes (e.g., 5) and fewer iterations (e.g., 30, 50) can be implemented to boost the running speed, whereas greater values for these parameters can be used to achieve better precision. There is also room to improve the score difference calculation to adjust the algorithm to users’ preferences. For instance, the scores can be compared in logarithm, so that the ratio of the two scores is taken instead of the difference. In addition, the reference datasets can be sampled from a uniform distribution over a box aligned to the principle components of the dataset, as an alternative option suggested in *Gap Statistics*^19^. Finally, it is highly advisable to always output the whole score curve and refer to it when choosing the optimal number of clusters. The trend in the curve can be helpful for understanding the overall clustering quality, and can provide insights into the inherent data structure.

## Additional Information

**How to cite this article**: Hu, C. W. *et al*. *Progeny Clustering:* A Method to Identify Biological Phenotypes. *Sci. Rep*. **5**, 12894; doi: 10.1038/srep12894 (2015).

## Supplementary Material

Supplementary Information

## Figures and Tables

**Table 1:** Parameter values used for *Progeny Clustering*.

Parameter	Value
Lower limit for the number of clusters: *K*_*min*_	2
Upper limit for the number of clusters: *K*_*max*_	10
Sampling size for each subset: 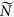	10
Number of sampling iterations: *R*	100
Number of reference datasets: *T*	10
Clustering method	*k-means*

**Table 2:** Performance comparison.

Dataset	Optimal K	*ProgenyClustering(score)*	*ProgenyClustering(gap)*	*Gap Statistics*	*Sihouette*	*Clest*	*Consensus Clustering*	*Model Explorer*
2-dimensional synthetic dataset	3	3	3	3	3	3	3	3
10-dimensional synthetic dataset	4	4	4	4	4	2	2	2
Iris dataset	3 (2)	2	5	8	2	3	2	2
Rat CNS dataset	6	10	6	7	5	5	2	2
cell phenotype dataset	4	4	4	5	4	4	3	6
AML adhesion pathway RPPA dataset	6 or 10	10	6	8	2	4	2	2

Supporting information for each method is included in the Supplement.
