# Supplementary material for: Progeny Clustering: A Method to Identify Biological Phenotypes
Source: Sci Rep. 2015 Aug 12;5:12894. doi: 10.1038/srep12894 (PMC4533525; doi:10.1038/srep12894)
Supplement: Supplementary Information [file srep12894-s1.pdf]

Supplementary Information
Progeny Clustering: A Method to Identify Biological Phenotypes

Chenyue W. Hu[1], Steven M. Kornblau[2], John W. Slater[3], and Amina A. Qutub[1*]

[1]Department of Bioengineering, Rice University
[2]Departments of Leukemia and Stem Cell Transplant, University of Texas MD Anderson Cancer Center
[3]Department of Biomedical Engineering, University of Delaware
[*]Correspondence to amina@rice.edu

Competing Methods

Gap Statistics

The total sum of within-class dissimilarity used in *Gap Statistics* [1] is defined as

$$W_K = \sum_{r=1}^{K} \frac{1}{2n_r} \sum_{i,j \in C_r} d_{ij}. \tag{1}$$

Here, d_{ij} denotes the dissimilarity between any of the two objects in the same cluster measured by *Euclidean distance*, and n_r is the number of objects in cluster C_r. The $\log W_K$ is then compared with its expectation through the definition of *Gap* as

$$Gap_K = \mathbb{E}[\log W_K] - \log W_K = \frac{1}{B} \sum_b \log W^*_{Kb} - \log W_K. \tag{2}$$

The expectation of $\log W_K$ is estimated by uniformly drawing B reference datasets either (a) over the range of each feature or (b) over a box aligned with principal components of the data. In our testing, the former prescription was implemented and 50 reference datasets were generated for each dataset. Let sd_K be the standard deviation and

$$s_K = sd_K \sqrt{1 + \frac{1}{B}}. \tag{3}$$

The optimal number of clusters $k_{optimal}$ is chosen via

$$K_{optimal} = \arg\min(Gap_K \geq Gap_{K+1} - s_{K+1}). \tag{4}$$

The *Gap* curves on all the datasets are illustrated in Figure S1, and the optimal number of clusters chosen from each curve is listed in Table 2.

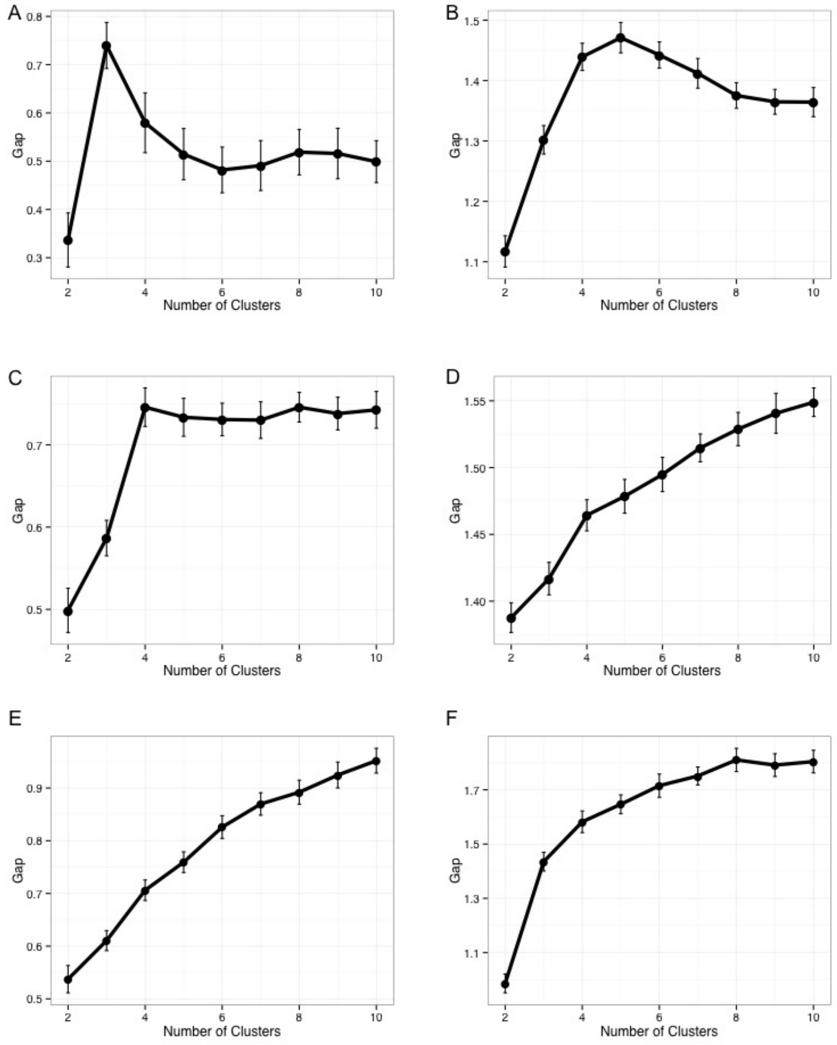

Figure S1: The *Gap Statistics* curves for the (A) 2-dimensional toy dataset; (B) 10-dimensional toy dataset; (C) cell phenotype dataset; (D) AML patient adhesion pathway RPPA dataset; (E) Iris dataset and (F) rat CNS dataset.

Silhouette

The *Silhouette* score [2] is defined as

$$s_{(i)} = \frac{b_{(i)} - a_{(i)}}{\max\{a_{(i)}, b_{(i)}\}}. \tag{5}$$

Here, $a_{(i)}$ is the average dissimilarity of i to all other members in the same cluster, and $b_{(i)}$ is the minimum of the average dissimilarity of i to members in another cluster. We used the default *Silhouette* function in Matlab to compute the score, in which dissimilarity is measured by *Euclidean distance*. The average *Silhouette* score was then taken as the quality measure for each number of clusters. The results from *Silhouette* for all the datasets are summarized in Table 2, and the *Silhouette* score curves are shown in Figure S2.

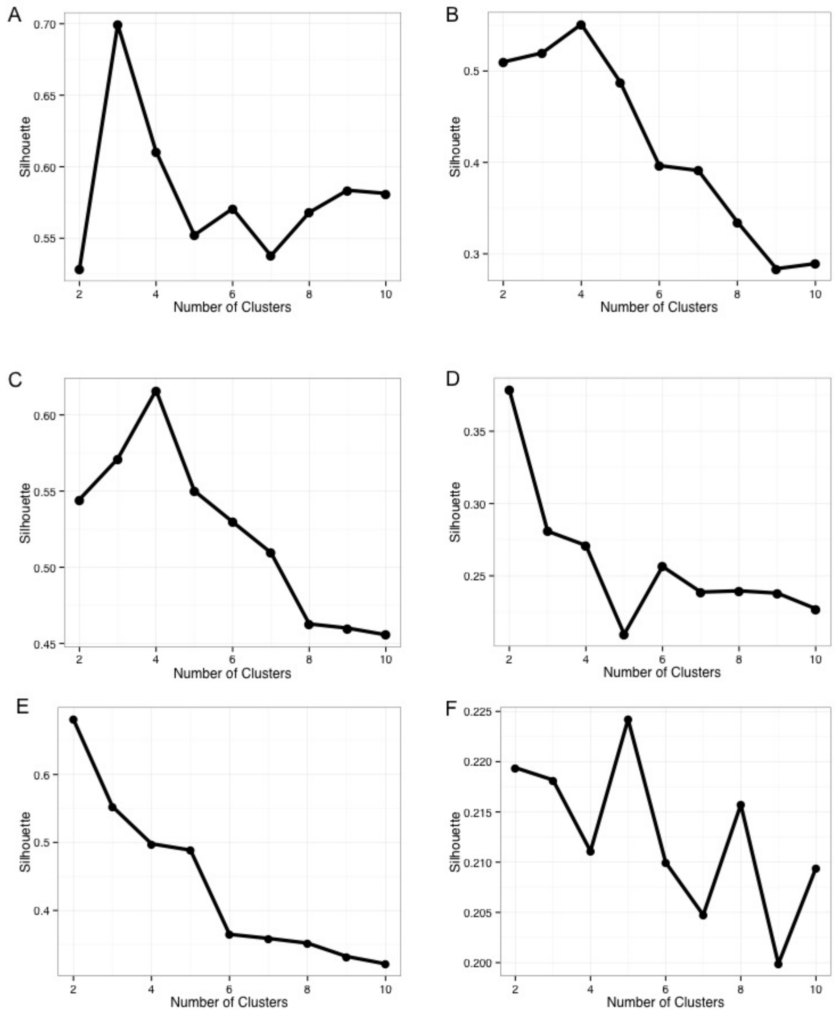

Figure S2: The *Silhouette* score curves for the (A) 2-dimensional toy dataset; (B) 10-dimensional toy dataset; (C) cell phenotype dataset; (D) AML patient adhesion pathway RPPA dataset; (E) Iris dataset and (F) rat CNS dataset.

Table S1: Parameter values used for *Clest*

**Parameter**	**Value**
Number of learning/test set iterations	20
Number of reference datasets	20
Size of learning sets	n/2
Clustering procedure	*k-means*
Classifier	Diagonal linear discriminant analysis
External index	Fowlkes and Mallows (FM)
Maximum *p-value* (p_{max})	0.05
Minimum difference statistic (d_{min})	0.05

Clest

The *Clest* algorithm [3] in brief follows the four general steps: (1) randomly splits the original dataset into a learning set and a test set; (2) builds a classifier by clustering the learning set; (3) applies both the classifier and clustering to the test set; (4) compares the two cluster assignments and outputs an external index t_K as the similarity statistic. Similar to *Gap Statistics*, reference datasets are also generated to estimate the expectation of the similarity statistic. The optimal number of clusters is defined as

$$K = \{2 \geq K \geq K_{max} : p_K \geq p_{max}, d_K \leq d_{min}\}, \quad (6)$$

in which d_K denotes the difference between the observed similarity statistic and its expected value, and p_K denotes the proportion of the reference similarity statistics that is greater than the observed value. The parameter values used for implementing *Clest* are listed in Table S1. The d_K curve for each dataset is shown in Figure S3, and the optimal number of clusters is shown in Table 2.

Consensus Clustering

The key component of *consensus clustering* [4] is the construction of consensus matrix, which is similar to the co-occurrence matrix in our method. The method repetitively resamples and clusters the dataset. The cluster assignments in different perturbed datasets are recorded in connectivity matrices, the sum of which is normalized by resampling frequencies, resulting in a final consensus matrix. To choose the optimal number of clusters, the method uses consensus distribution to assess how the entries of the consensus matrix are distributed within the range from 0 to 1. A clean matrix, representing a good clustering, would have a consensus distribution concentrated at 0 and 1. The *empirical cumulative distribution* (CDF) is defined as

$$CDF(c) = \frac{\sum_{i<j} 1\{M_{(i,j)} \leq c\}}{\frac{N(N-1)}{2}}, \quad (7)$$

where $M_{(i,j)}$ denotes the entry (i, j) of the consensus matrix M, N denotes the number of rows in M, and $1\{\ldots\}$ is an indicator function. The optimal number K is chosen based on the highest $\triangle(K)$ defined as

$$\triangle(K) = \begin{cases} A(K), & K = 2 \\ \frac{A(K)-A(K-1)}{A(K-1)}, & K > 2 \end{cases}, \quad (8)$$

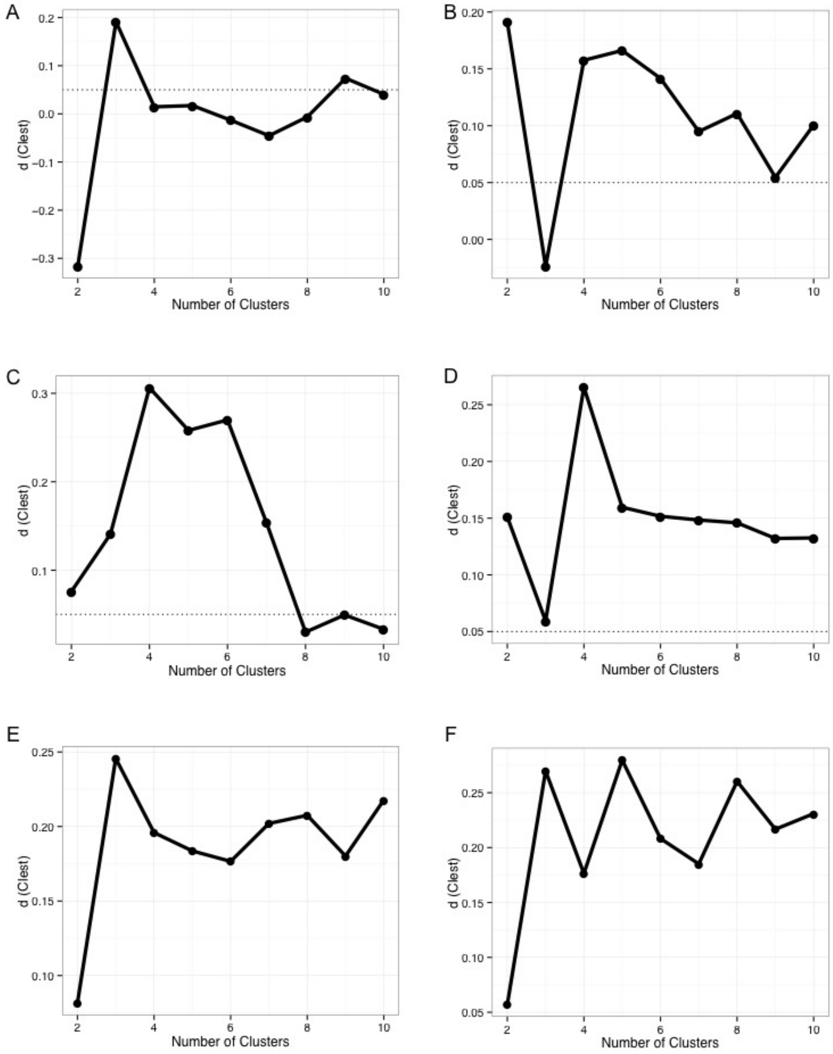

Figure S3: Results from *Clest* for the (A) 2-dimensional toy dataset; (B) 10-dimensional toy dataset; (C) cell phenotype dataset; (D) AML patient adhesion pathway RPPA dataset; (E) Iris dataset and (F) rat CNS dataset.

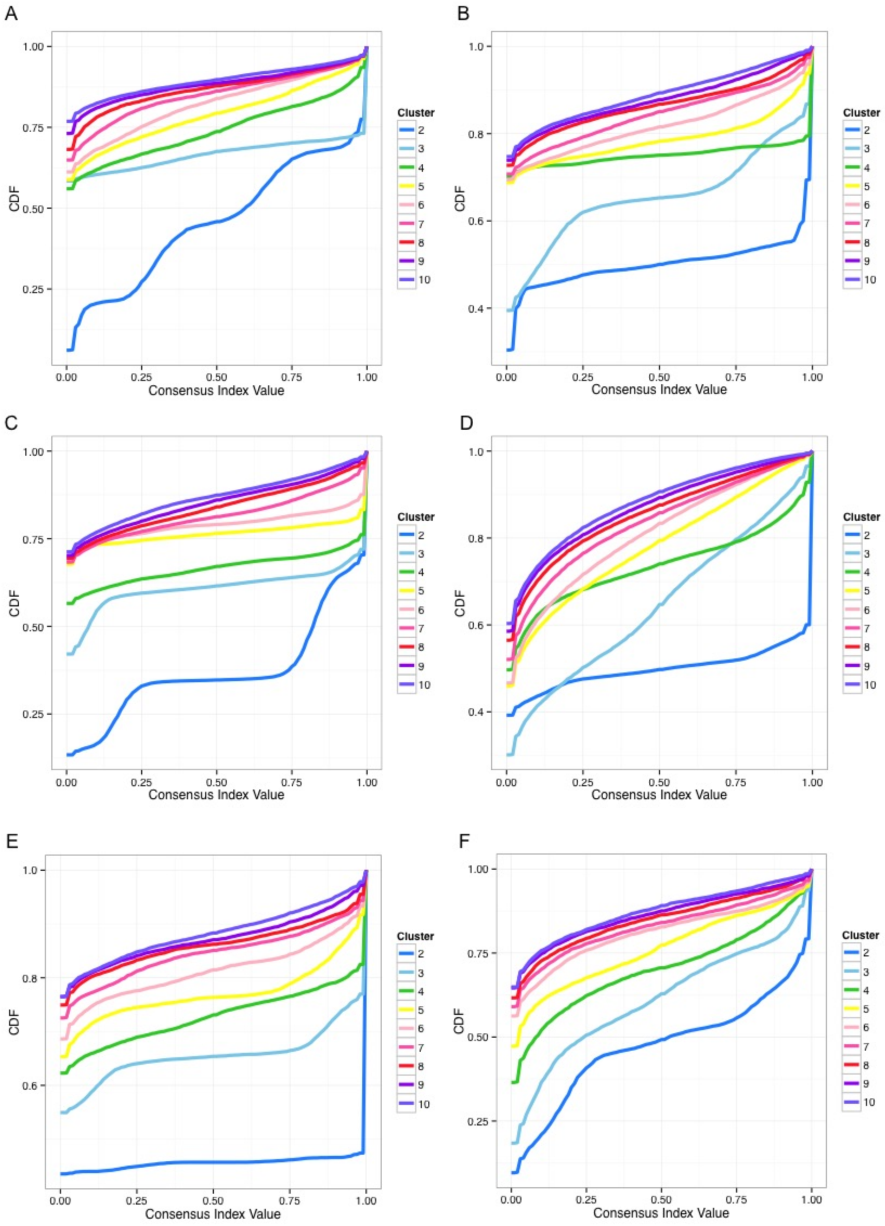

Figure S4: The CDF curves from *Consensus Clustering* for the (A) 2-dimensional toy dataset; (B) 10-dimensional toy dataset; (C) cell phenotype dataset; (D) AML patient adhesion pathway RPPA dataset; (E) Iris dataset and (F) rat CNS dataset.

in which $A(K)$ is the area under the CDF for k-cluster partition of the data. For implementation, we resampled the data 100 times to construct the consensus matrix. Both the CDF and $\triangle(K)$ curves were plotted (Figure S4 and S5), and the optimal number K for each dataset is shown in Table 2.

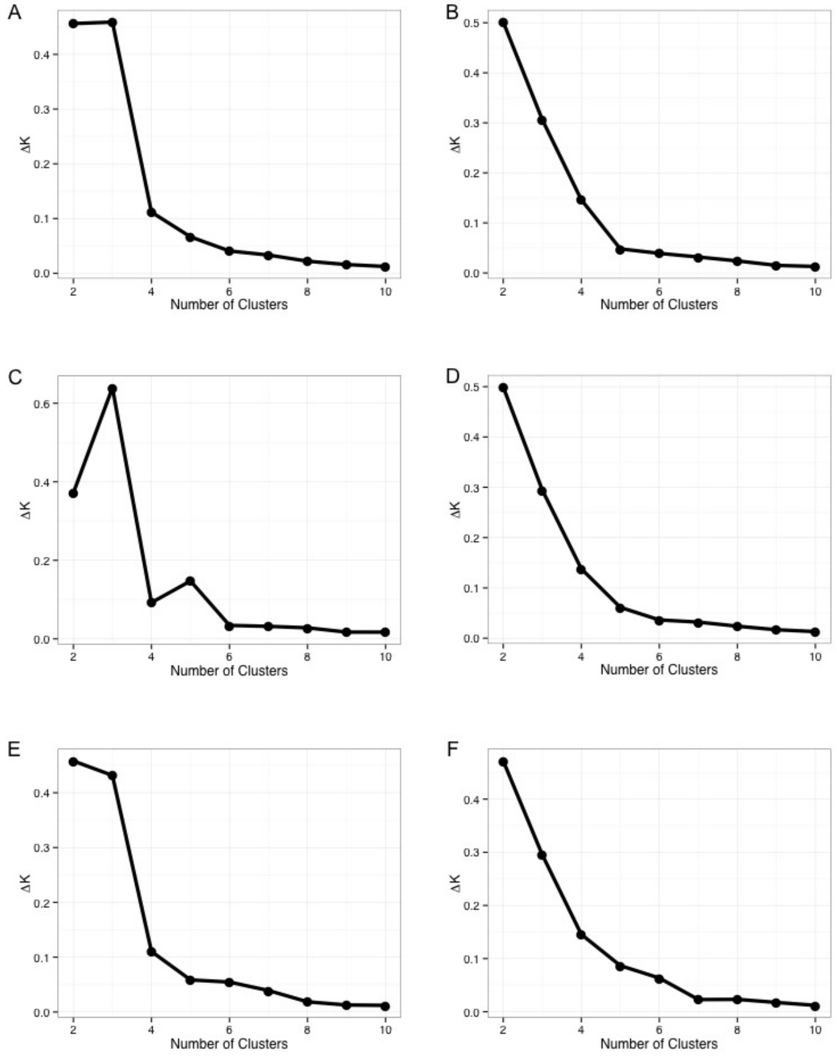

Figure S5: The $\triangle K$ curves from *Consensus Clustering* for the (A) 2-dimensional toy dataset; (B) 10-dimensional toy dataset; (C) cell phenotype dataset; (D) AML patient adhesion pathway RPPA dataset; (E) Iris dataset and (F) rat CNS dataset.

Model Explorer

The *Model Explorer* method [5] first generates two sub-samples (L_1, L_2) with a ratio f of the original dataset, $f \in (0.5, 1)$. The two sub-samples are then clustered separately, and the cluster assignments are compared at the intersection of the two sets by a specified similarity measure. Similar to *Consensus Clustering*, the optimal number of clusters is chosen based on the *empirical cumulative distribution* of the similarity measure. Here, we chose 0.8 for f, and used the *Jaccard coefficient* as the similarity measure, which is defined as

$$J(L_1, L_2) = \frac{< C^1, C^2 >}{< C^1, C^2 > + < C^2, C^2 > - < C^1, C^2 >}. \tag{9}$$

The C^1 and C^2 are co-occurrence matrices for the intersection under two cluster assignments, and $<, >$ represents the dot product of two matrices. The procedure was repeated 100 times, and the cumulative distribution curves of similarity were plotted (Figure S6) for finding the optimal K (Table 2).

Experimental Methods

Cell Phenotype Dataset

The cell phenotype dataset is derived from high content image analysis of 444 microscope images of single primary human umbilical vein endothelial cells. Four of these images were cells of interest (COI), that were cultured on fibronectin-coated glass coverslips, fixed, fluorescently immunolabeled for the cytoskeletal proteins vinculin and actin, and stained by DAPI for nuclei. The remaining cells were cultured on pattern configurations derived from the 4 COIs which were created via Image Guided Laser Scanning Lithography (IG-LSL).

The process of patterning via LSL is described in detail elsewhere [6]. Briefly, it consists of four steps: (1) the image of the COI is processed to create a virtual mask, that defines regions of interest (ROIs) to control the laser position and shutter during Laser Scanning Lithography; (2) a focused 532 nm laser is used to photothermally desorb ROIs of an oligo(ethylene glycol) (OEG) terminated alkanethiol from a platinum-coated glass coverslip; (3) the surface is exposed to a solution of fibronectin that adsorbs exclusively to the bare platinum patterns where the OEG was removed; and (4) endothelial cells are seeded onto the surfaces. In total, four main categories of cells were patterned from the four original COIs, containing 102 crescent-shaped cells, 123 star-shaped cells, 179 square-shaped cells, and 40 Texas-shaped cells (Figure 4). There were also two subcategories of cells under each main category (Figure S7) generated by different virtual masks from each COI: one used a single, continuous pattern derived from an outline of the COI, and the other used a discontinuous pattern composed of many individual features derived from an image of each COIs vinculin containing adhesion sites.

To ascertain the efficiency of this biomimetic nanolithography patterning and to identify common structural properties of endothelial cells, high content image analysis was performed on images of the four COIs and their respective patterned cells. This analysis is described in detail elsewhere [7]. Briefly, it provides a means to automatically obtain a suite of morphology metrics of single cells from images. 41 morphology metrics were applied to cells in this dataset. Metrics fell into six main categories: (1) contouring; (2) texture; (3) polarity; (4) adhesion sites; (5) intensity, area and shape; and (6) fiber alignment. *K-means* cluster analysis was then performed on the total population of 444 cells (each described by a set of 41 metrics) to determine how cytoskeletal and nuclei features of the patterned cells aligned with the COIs.

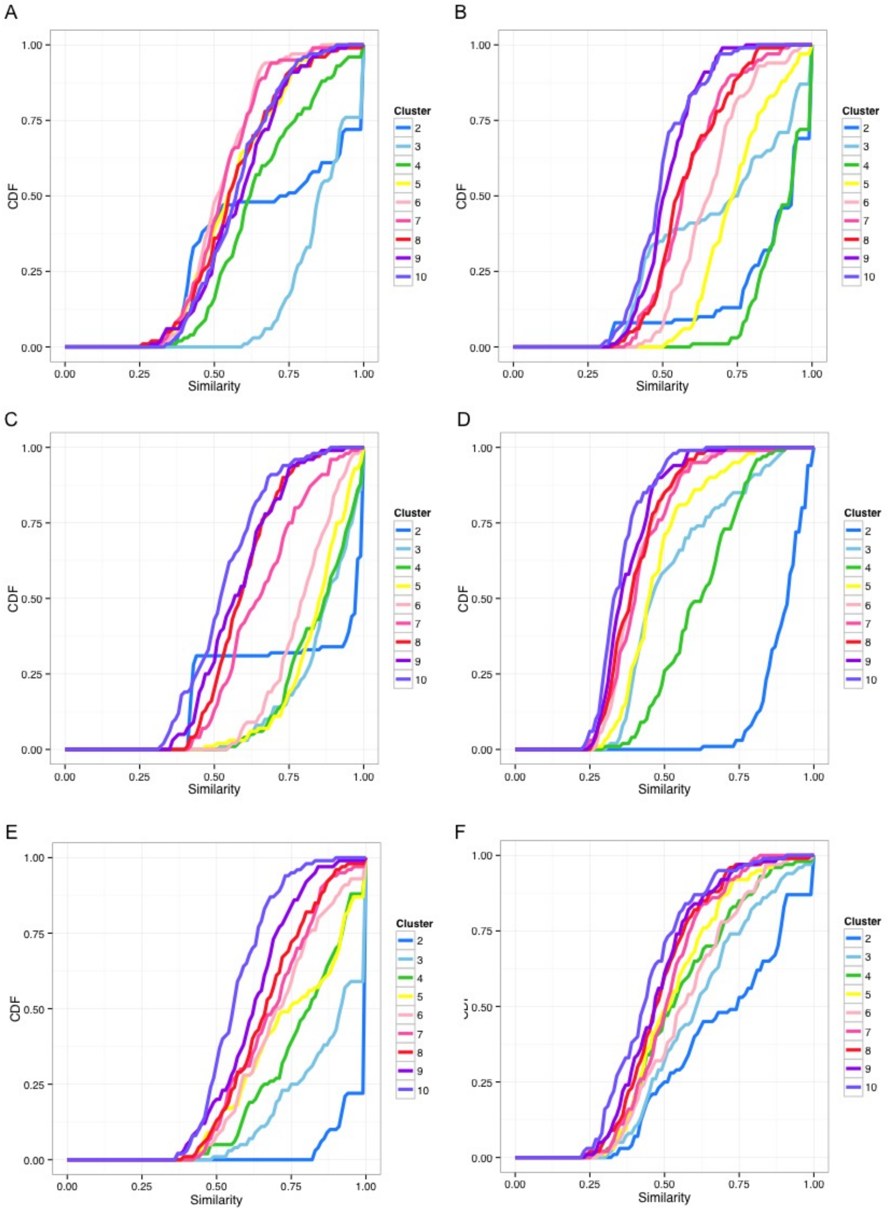

Figure S6: The CDF curves from *Model Explorer* for the (A) 2-dimensional toy dataset; (B) 10-dimensional toy dataset; (C) cell phenotype dataset; (D) AML patient adhesion pathway RPPA dataset; (E) Iris dataset and (F) rat CNS dataset.

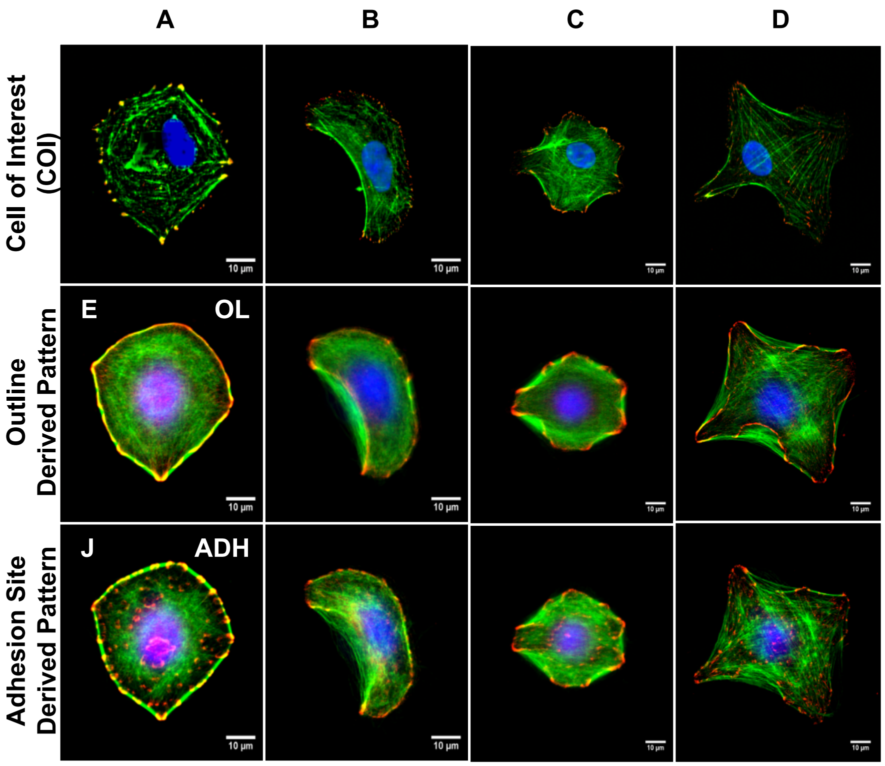

Figure S7: Images of (1st row) 4 COIs; (2nd row) 4 subcategories patterned by outline from 4 COIs; (3rd row) 4 subcategories patterned by adhesion sites from 4 COIs.

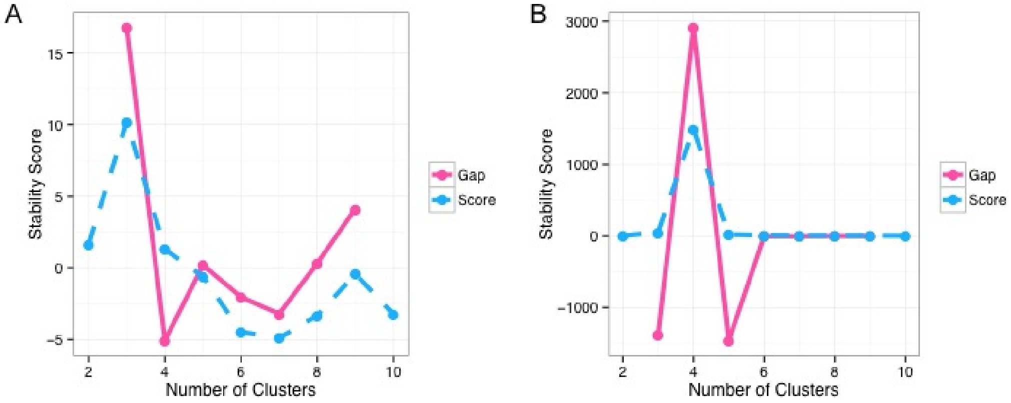

Figure S8: *Progeny Clustering* results for (A) three-cluster two-dimensional synthetic dataset and (B) four-cluster ten-dimensional synthetic dataset using *Hierarchical Clustering*. The results are consistent with that using *k-means* (Figure 2 and 3).

AML Adhesion Pathway RPPA Dataset

Expression levels of 10 adhesion pathway proteins were obtained from 560 acute myeloid leukemia (AML) samples collected by researchers at the University of Texas MD Anderson Cancer Center from patient blood, marrow and plasma. The 10 adhesion-related proteins were chosen based on their hypothesized biological activity in leukemia and significance in determining patient survival outcome. Reverse phase protein array (RPPA) technology was employed to obtain these protein levels. In RPPA, an arrayer spotted whole cell lysates onto nitrocellulose slides in serial dilution, and each slide was probed with a highly validated antibody against a protein (total, phospho or cleaved) of interest [8]. The overall positive control lysate consisted of a pooled mixture of 11 AML cell lines, which were used to account for variations in staining, background and loading, while the lysate buffer served as the negative control. MicroVigene software was used to scan and digitize slides, and the data was normalized following established protocols [8, 9].

References

[1] Tibshirani, R., Walther, G. & Hastie, T. Estimating the number of clusters in a data set via the gap statistic. *Journal of the Royal Statistical Society: Series B (Statistical Methodology)* **63**, 411–423 (2001).

[2] Rousseeuw, P. J. Silhouettes: a graphical aid to the interpretation and validation of cluster analysis. *Journal of computational and applied mathematics* **20**, 53–65 (1987).

[3] Dudoit, S. & Fridlyand, J. A prediction-based resampling method for estimating the number of clusters in a dataset. *Genome biology* **3** (2002).

[4] Monti, S., Tamayo, P., Mesirov, J. & Golub, T. Consensus clustering: a resampling-based method for class discovery and visualization of gene expression microarray data. *Machine learning* **52**, 91–118 (2003).

[5] Ben-Hur, A., Elisseeff, A. & Guyon, I. A stability based method for discovering structure in clustered data. In *Pacific symposium on biocomputing*, vol. 7, 6–17 (2001).

[6] Slater, J. H., Miller, J. S., Yu, S. S. & West, J. L. Fabrication of multifaceted micropatterned surfaces with laser scanning lithography. *Advanced Functional Materials* **21**, 2876–2888 (2011).

[7] Ryan, D. *et al.* An automated method for classifying and matching endothelial cell cytoskeletal and signaling phenotypes. *To be submitted* (2013).

[8] Tibes, R. *et al.* Reverse phase protein array: validation of a novel proteomic technology and utility for analysis of primary leukemia specimens and hematopoietic stem cells. *Molecular cancer therapeutics* **5**, 2512–2521 (2006).

[9] Kornblau, S. M. *et al.* Functional proteomic profiling of aml predicts response and survival. *Blood* **113**, 154–164 (2009).
